# Cytokines and Chemokines Are Detectable in Swivel-Derived Exhaled Breath Condensate (SEBC): A Pilot Study in Mechanically Ventilated Patients

**DOI:** 10.1155/2020/2696317

**Published:** 2020-01-11

**Authors:** Philip van der Zee, Inez van Walree, Jan-Willem Fijen, Arend-Jan van Houte, Heleen van Velzen-Blad, Ger Rijkers, Diederik Gommers, Henrik Endeman

**Affiliations:** ^1^Department of Adult Intensive Care, Erasmus Medical Center, Doctor Molewaterplein 40, 3015 GD Rotterdam, Netherlands; ^2^Department of Adult Intensive Care, Diakonessenhuis, Bosboomstraat 1, 3582 KE Utrecht, Netherlands; ^3^Department of Medical Microbiology and Immunology, St. Antonius Ziekenhuis, Koekoekslaan 1, 3435 CM Nieuwegein, Netherlands; ^4^Department of Medical Microbiology and Clinical Chemistry, Diakonessenhuis, Bosboomstraat 1, 3582 KE Utrecht, Netherlands; ^5^Science Department, University College Roosevelt, Lange Noordstraat 1, 4331 CB Middelburg, Netherlands

## Abstract

**Introduction:**

Exhaled breath condensate (EBC) is a noninvasive method to collect samples from the respiratory tract. Usually, a thermoelectric cooling module is required to collect sufficient EBC volume for analyses. In here, we assessed the feasibility of cytokine and chemokine detection in EBC collected directly from the ventilator circuit without the use of a cooling module: swivel-derived exhaled breath condensate (SEBC).

**Methods:**

SEBC was prospectively collected from the swivel adapter and stored at -80°C. The objective of this study was to detect cytokines and chemokines in SEBC with a multiplex immunoassay. Secondary outcomes were to assess the correlation between cytokine and chemokine concentrations in SEBC and mechanical ventilation parameters, systemic inflammation parameters, and hemodynamic parameters.

**Results:**

Twenty-nine SEBC samples were obtained from 13 ICU patients. IL-1*β*, IL-4, IL-8, and IL-17 were detected in more than 90% of SEBC samples, and significant correlations between multiple cytokines and chemokines were found. Several significant correlations were found between cytokines and chemokines in SEBC and mechanical ventilation parameters and serum lactate concentrations.

**Conclusion:**

This pilot study showed that it is feasible to detect cytokines and chemokines in SEBC samples obtained without a cooling module. Despite small sample size, correlations were found between cytokines and chemokines in SEBC and mechanical ventilation parameters, as well as serum lactate concentrations. This simple SEBC collection method provides the opportunity to collect EBC samples in large prospective ICU cohorts.

## 1. Introduction

Pulmonary inflammation is the hallmark of acute respiratory distress syndrome (ARDS) and ventilator-associated pneumonia (VAP) [1, 2]. Consecutive measurements of pulmonary inflammation could identify mechanically ventilated patients that develop ARDS or VAP in an early phase of the disease or even patients at risk.

Bronchoscopy with bronchoalveolar lavage is an invasive method that is used to directly detect pulmonary inflammation. However, a bronchoscopy is not routinely performed until clinical or radiological symptoms of ARDS or VAP have developed. Exhaled breath condensate (EBC) is a noninvasive method to sample the airway lining fluid that covers the respiratory tract [3–5]. A variety of inflammatory biomarkers has been detected in EBC [6, 7]. In mechanically ventilated patients, EBC samples are collected by guiding exhaled breath air through a thermoelectric cooling module using additional tubing. Cooling down exhaled breath air is required to collect sufficient sample volume for analyses [8]. The necessity for a cooling module and additional mechanical ventilation tubing prevented the collection of EBC in large prospective cohorts at the intensive care unit (ICU), as it is both complex and time-consuming.

Multiplex immunoassays are able to detect cytokines and chemokines in very small sample volumes. A volume of 50 microliters is sufficient to obtain reliable results. In all patients on mechanical ventilation with a heat and moisture exchanger (HME), a small volume of EBC cumulates in the expiratory tubing of the ventilatory circuit: swivel-derived exhaled breath condensate (SEBC).

The hypothesis of this study was that it was feasible to detect cytokines and chemokines in SEBC obtained from mechanically ventilated ICU patients. We collected SEBC material directly from the ventilator circuit and used a multiplex immunoassay to detect cytokines and chemokines.

## 2. Methods

### 2.1. Study Design and Setting

This prospective observational pilot study was performed in the ICU of Diakonessenhuis, Utrecht, the Netherlands. Adult patients on invasive mechanical ventilation were included in this study. We excluded patients with purulent or haemorrhagic sputum that required active humidification instead of a heat and moisture exchanger (HME). The study was approved by the medical ethical committee (METC) of the Diakonessenhuis Utrecht. A waiver for informed consent was given due to the noninvasive nature of the study.

### 2.2. Study Outcomes

The primary outcome of this feasibility study was to detect cytokines and chemokines in SEBC obtained in standard ICU bedside conditions. Secondary outcomes were to assess the correlation between cytokine and chemokine concentrations in SEBC and mechanical ventilation parameters, systemic inflammation parameters, and hemodynamic parameters.

### 2.3. Data Collection

#### 2.3.1. Swivel-Derived EBC Sample Collection

SEBC sampling was performed by two researchers (HE and IW) between 8 and 10 a.m. before routine airway care. The tube and the swivel adapter were disconnected, and droplets in the swivel adapter were directly aspirated with a 3 mL disposable pipet. The SEBC samples were stored in 2 mL containers and immediately cooled in ice. Within 15 minutes following collection, the samples were stored at -80°C.

#### 2.3.2. Multiplex Immunoassay

SEBC samples were analysed with a multiplex immunoassay (Luminex, Austin, TX, R&D Systems Cytokines) according to manufacturer's instruction protocol (R&D Systems). The concentrations of a set of cytokines (IL-1*β*, IL-2, IL-4, IL-5, IL-6, IL-7, IL-8, IL-10, IL-12, IL-13, IL-17, IFN-*γ*, G-CSF, and TNF-*α*) and chemokines (MCP-1 and MIP-1*β*) were measured.

#### 2.3.3. Patient Data Collection

Demographic and clinical characteristics of the patients were retrieved from the patient data management system (PDMS, MetaVision). All patients were ventilated with a Servo-i mechanical ventilator in either pressure control or pressure support mode. Airway pressure levels and fraction of inspired oxygen (FiO_2_) were adjusted by the attending physician. The following respiratory variables were recorded at the moment of SEBC sampling: mode of ventilation, tidal volume (Vt), positive end-expiratory pressure (PEEP), peak airway pressure, plateau airway pressure, FiO_2_, pulmonary dynamic compliance, and PaO_2_/FiO_2_ (P/F) ratio. In addition, the mean arterial blood pressure (MAP), heart rate, urinary output in two hours before sampling, dose of noradrenaline (target MAP > 65 mmHg), and central temperature were recorded. The following laboratory parameters were assessed at the day of SEBC sampling: serum lactate, haemoglobin, sodium, potassium, CRP, white blood cell count (WBC), platelet count, urea, and creatinine. Arterial blood gas results before the moment of sampling were recorded as well.

### 2.4. Statistical Analysis

Descriptive characteristics are shown as the median and range. Correlation coefficients were calculated by using Pearson's correlation coefficients, provided that at least 10 samples had detectable cytokine or chemokine concentrations. All statistical analyses were performed in IBM SPSS Statistics 21. A difference of *p* < 0.05 was considered statistically significant.

## 3. Results

### 3.1. Patient Characteristics

Twenty-nine SEBC samples were obtained from 13 patients (median 2 samples, range 1-4). Patient characteristics are shown in Table 1. None of the patients was diagnosed with a VAP or ARDS according to recent definitions [1, 9]. Seven SEBC samples were taken during pressure control ventilation and 22 during pressure support ventilation.

### 3.2. Feasibility

All cytokines and chemokines were detectable in SEBC samples except for IL-2 (Table 2). IL-1*β*, IL-4, IL-8, and IL-17 were detected in more than 90% of SEBC samples, but ranges varied greatly. In addition, we found significant correlations between cytokine and chemokine concentrations (Table 3).

Correlations with mechanical ventilation parameters, systemic inflammation, and hemodynamic parameters are shown in the supplementary files ([Supplementary-material supplementary-material-1]). Cytokine and chemokine concentrations in SEBC samples did not differ between patients on pressure control or pressure support ventilation. High Vt (mL/kg PBW) was correlated with IL-10 (*r* = .391, *p* < 0.05), IL-12 (*r* = .392, *p* < 0.05), and MIP-1*β* (*r* = .397, *p* < 0.05). High P/F ratio was correlated with IL-8 (*r* = .427, *p* < 0.05) and MCP-1 (*r* = .381, *p* < 0.05). In addition, we observed significant correlations between high serum lactate and IL-1*β* (*r* = .889, *p* < 0.01), IL-6 (*r* = .817, *p* < 0.01), IL-8 (*r* = .742, *p* < 0.05), MIP-1*β* (*r* = .797, *p* < 0.01), and TNF-*α* (*r* = .790, *p* < 0.01).

## 4. Discussion

In this pilot study, we showed that it is feasible to detect cytokines and chemokines in SEBC samples obtained directly from the ventilator circuit without the use of a cooling module. The cytokines and chemokines in SEBC correlated significantly with each other. We found correlations between cytokine and chemokine concentrations and mechanical ventilation parameters, as well as high serum lactate. Although the small sample size of this study prevents any definitive conclusions, the measurement of cytokines and chemokines in SEBC has the potential to become a noninvasive bedside method to detect pulmonary inflammation.

In line with a previous research, cytokine and chemokine concentrations in SEBC samples were low and concentrations varied widely [10–15]. Both the wide variation and low concentrations are the result of EBC formation in the airways and EBC sample dilution. The exact origin of EBC is uncertain, but McNeil et al. found that there is a correlation between fluids extracted from the HME filter and oedema fluid aspirated directly from the airways [16]. Therefore, it is suggested that EBC originates from the airway lining fluid covering the respiratory tract [8, 17]. Up to 99.9% of EBC consists of evaporated water, and only a small proportion consists of both volatile and nonvolatile compounds [3, 18–20]. The nonvolatile compounds, including cytokines and chemokines, are shed from the airway surfaces as small droplets of airway lining fluid during tidal breathing [8, 17, 19]. The number of droplets detected in exhaled breath air varies greatly resulting in variable sample dilution [17]. Currently, there is no consensus on a method to correct for sample dilution. It has been suggested to calculate proportions between substances in order to correct for sample dilution [21, 22]. In our study, we found multiple well-known correlations between cytokines and chemokines in SEBC samples. The combination of IL-6 and IL-8 is frequently used in ARDS research [23, 24], whereas IL-13 was only present in combination with high IL-5 concentrations; both are T-helper cell 2 cytokines associated with airway hyperresponsiveness [25].

Mechanical ventilation with high airway pressures and high tidal volumes is associated with increased mortality in patients with ARDS [26]. Until now, only Gessner and Hartmut found a strong correlation between EBC nitrite levels and tidal volume in an ICU population [27]. In this study, we found significant correlations between high peak airway pressure and G-CSF and between high tidal volume (mL/kg PBW) and IL-10, IL-12, and MIP-1*β*. In contrast, Fernandez-Bustamante et al. did not find a difference in EBC cytokines between low tidal volume (6 mL/kg) and intermediate tidal volume (10 mL/kg) after one hour of mechanical ventilation in healthy perioperative patients [15]. Multiple cytokines were undetectable in their study despite the use of a multiplex immunoassay. The undetectable concentrations could be explained by the small difference in tidal volumes, as a tidal volume of 10 mL/kg does not increase the mortality rate in ICU patients without ARDS [28]. In addition, in healthy perioperative patients, the endothelial barrier function is preserved and might not have been affected by one hour of mechanical ventilation.

Despite the small sample size, we found strong correlations between high serum lactate and IL-1*β*, IL-6, IL-8, G-CSF, MIP-1*β*, and TNF-*α* concentrations in SEBC. Previously, we have suggested that ATP levels in EBC samples did not accurately reflect serum lactate in healthy patients [29]. In critically ill patients, the endothelial barrier function is impaired, which could be an explanation for the strong correlations found between serum lactate and cytokines and chemokines in SEBC. Parameters associated with an active infection, such as CRP and white blood cell count, were not significantly correlated with cytokines and chemokines in SEBC samples. Therefore, we hypothesize that the raised concentrations of cytokines and chemokines in SEBC samples are the result of endothelial barrier dysfunction, and not of infection.

This pilot study has several limitations. First, this study was designed as a pilot study with a primary aim to establish the feasibility of detection of cytokines and chemokines in SEBC samples collected without a cooling module. Therefore, the sample size was small and the correlations found are likely to overestimate or underestimate the true effect. Second, we did not collect matching plasma samples to measure cytokines and chemokines to correlate with the SEBC samples. Third, no SEBC dilution factors were calculated. We decided to present the raw data as there is no consensus on the calculation of EBC sample dilution [3, 5]. IL-8 could be used as a denominator, as this cytokine is detectable in 96% of measured samples. Lastly, we did not compare SEBC with EBC collected by a cooling module, as there is no gold standard for EBC collection.

Despite these limitations, this pilot study demonstrated that cytokines and chemokines can be detected in SEBC. The only prerequisite is the use of a HME, as active humidification potentially results in sample dilution [5]. This simple SEBC collection method provides a unique opportunity to collect EBC samples in large prospective ICU cohorts, in order to determine whether cytokines and chemokines are correlated with mechanical ventilation parameters and systemic parameters or even predict the development of ARDS or VAP.

## 5. Conclusion

This pilot study showed that it is feasible to detect cytokines and chemokines in SEBC samples obtained directly from the ventilator circuit without the use of a cooling module. Although the sample size was small, correlations were found between cytokines and chemokines in SEBC, as well as mechanical ventilation parameters and high serum lactate concentrations. This simple SEBC collection method provides the opportunity to collect EBC samples in large prospective ICU cohorts.

## Figures and Tables

**Table 1:** Patient characteristics of 13 mechanically ventilated patients.

Male (*n*, %)	7 (54)
Age	69 (37-77)
Reason for admission, *n* (%)	
(i) Complications of previous abdominal or vascular surgery	4 (30%)
(ii) Cardiac failure	2 (15%)
(iii) Pneumonia (without severe sepsis or shock)	2 (15%)
(iv) Severe sepsis/septic shock	2 (15%)
(v) Other	3 (23%)
APACHE II score	25 (14-39)
Peak airway pressure (cmH_2_O)	18 (10-36)
Plateau airway pressure (cmH_2_O)	12.5 (6-24)
Positive end-expiratory pressure (cmH_2_O)	8 (5-18)
Tidal volume (mL/kg predicted body weight)	7.2 (5.1-11.0)
Fraction of inspired oxygen (%)	35 (25-70)
PaO_2_/FiO_2_ ratio (mmHg)	252 (95-364)
Respiratory system compliance (mL/cmH_2_O)	48 (8-208)

Data are presented as the median and range unless stated otherwise.

**Table 2:** Concentrations of cytokines and chemokines in SEBC (*n* = 29).

Cytokine/chemokine	Detection, *n* (%)	Median (pg/mL)	Range (pg/mL)
IL-1*β*	26 (93)^∗^	0.12	0.00-13.71
IL-2	0	—	—
IL-4	26 (90)	0.26	0.00-0.84
IL-5	3 (10)	0.00	0.00-0.60
IL-6	15 (52)	0.00	0.00-131.80
IL-7	2 (7)	0.00	0.00-9.37
IL-8	27 (96)^∗^	2.55	0.00-6448.00
IL-10	24 (86)^∗^	0.03	0.00-61.14
IL-12	21 (72)	0.04	0.00-7.36
IL-13	1 (3)	0.00	0.00-1.52
IL-17	26 (90)	1.30	0.00-4.75
G-CSF	2 (7)^∗^	0.00	0.00-17.90
IFN-*γ*	12 (41)	0.00	0.00-96.13
MCP-1	25 (86)	0.86	0.00-711.76
MIP-1*β*	16 (55)	0.22	0.00-656.92
TNF-*α*	12 (41)	0.00	0.00-15.51

^∗^One result not available in immunoassay (*n* = 28). IL: interleukin; G-CSF: granulocyte colony-stimulating factor; IFN: interferon; MCP: monocyte chemoattractant protein; MIP: macrophage inflammatory protein; TNF: tumour necrosis factor.

**Table 3:** Correlation coefficients between cytokines and chemokines in SEBC.

	IL-1*β*	IL-4	IL-6	IL-8	IL-10	IL-12	IL-17	IFN-*γ*	MCP-1	MIP-1*β*	TNF -*α*
IL-1*β*	—										
IL-4	.021	—									
IL-6	.212	-.063	—								
IL-8	.097	-.156	.963^∗∗^	—							
IL-10	-.089	-.122	-.045	-.048	—						
IL-12	-.071	-.100	-.027	-.040	.991^∗∗^	—					
IL-17	.076	.115	-.164	-.212	.257	-.212	—				
IFN-*γ*	-.103	.056	-.062	-.095	.823^∗∗^	.822^∗∗^	.063	—			
MCP-1	.060	-.128	.967^∗∗^	.990^∗∗^	.045	.058	-.220	-.001	—		
MIP-1*β*	-.082	-.116	-.006	-.009	.999^∗∗^	.992^∗∗^	-.278	.821^∗∗^	.084	—	
TNF-*α*	.352	.336	.365	.213	-.02	.024	.282	.411^∗^	.228	.017	—

A positive value indicates a positive correlation, whereas a negative value indicates a negative correlation; ^∗^*p* < 0.05 and ^∗∗^*p* < 0.01. IL: interleukin; IFN: interferon; MCP: monocyte chemoattractant protein; MIP: macrophage inflammatory protein; TNF: tumour necrosis factor.

## Data Availability

The datasets used and/or analysed during the current study are available from the corresponding author on reasonable request.

## Abbreviations

ARDS: Acute respiratory distress syndrome
EBC: Exhaled breath condensate
FiO_2_: Fraction of inspired oxygen
G-CSF: Granulocyte colony-stimulating factor
HME: Heat and moisture exchanger
ICU: Intensive care unit
IFN: Interferon
IL: Interleukin
MAP: Mean arterial blood pressure
MCP: Monocyte chemoattractant protein
MIP: Macrophage inflammatory protein
METC: Medical ethical committee
PEEP: Positive end-expiratory pressure
P/F: PaO_2_/FiO_2_
SEBC: Swivel-derived exhaled breath condensate
TNF: Tumour necrosis factor
VAP: Ventilator-associated pneumonia
Vt: Tidal volume
WBC: White blood cell count.

## Supplementary Materials

Supplementary Materials Table 1: correlation coefficients between mechanical ventilation parameters and concentrations of cytokines and chemokines in SEBC. Table 2: correlation coefficients between parameters of systemic inflammation and concentrations of cytokines and chemokines in SEBC. Table 3: correlation coefficients between parameters of circulation and concentrations of cytokines and chemokines in SEBC.
