# Supplementary material for: Cytokines and Chemokines Are Detectable in Swivel-Derived Exhaled Breath Condensate (SEBC): A Pilot Study in Mechanically Ventilated Patients
Source: Dis Markers. 2020 Jan 11;2020:2696317. doi: 10.1155/2020/2696317 (PMC6977328; doi:10.1155/2020/2696317)
Supplement: Supplementary Materials — Table 1: correlation coefficients between mechanical ventilation parameters and concentrations of cytokines and chemokines in SEBC. Table 2: correlation coefficients between parameters of systemic inflammation and concentrations of cytokines and chemokines in SEBC. Table 3: correlation coefficients between parameters of circulation and concentrations of cytokines and chemokines in SEBC. [file 2696317.f1.docx]

**Supplementary files**

**Table 1** Correlation coefficients between mechanical ventilation parameters and concentrations of cytokines and chemokines in SEBC

|  | PEEP | Ppeak | Pplat | Compliance | FiO_2_ (%) | P/F | Vt/kg |
| --- | --- | --- | --- | --- | --- | --- | --- |
| IL-1β | .016 | .208 | .011 | -.107 | .195 | -.023 | -.166 |
| IL-4 | -.162 | -.051 | .234 | .068 | .019 | -.063 | -.089 |
| IL-6 | .003 | .013 | -.055 | .004 | .033 | .331 | .092 |
| IL-8 | -.006 | -.129 | -.143 | .077 | -.071 | .427* | .172 |
| IL-10 | .003 | -.190 | -.160 | .265 | .024 | .005 | .391* |
| IL-12 | .042 | -.170 | -.101 | .298 | .081 | -.028 | .392* |
| IL-17 | -.098 | -.069 | -.025 | .072 | .076 | -.160 | -.129 |
| IFN-γ | .092 | -.019 | .059 | .213 | .096 | -.173 | .236 |
| MCP-1 | .032 | -.105 | -.098 | .100 | -.036 | .381* | .195 |
| MIP-1β | -.006 | -.197 | -.162 | .267 | .021 | .031 | .397* |
| TNF-α | -.033 | .288 | .207 | -.175 | .172 | -.082 | .230 |

A positive value indicates a positive correlation, whereas a negative value indicates a negative correlation, * < 0.05 ** < 0.01. Abbreviations: IL interleukin, G-CSF granulocyte colony-stimulating factor, IFN interferon, MCP monocyte chemoattractant protein, MIP macrophage inflammatory protein, TNF tumour necrosis factor, PEEP positive end-expiratory pressure, Ppeak peak airway pressure, Pplat plateau airway pressure, FiO_2_ fraction of inspired oxygen, P/F PaO_2_/FiO_2_ ratio, Vt tidal volume.

**Table 2** Correlation coefficients between parameters of systemic inflammation and concentrations of cytokines and chemokines in SEBC

|  | Temperature | CRP | Thrombocytes | Leukocytes | Haemoglobin | Urea | Creatinine |
| --- | --- | --- | --- | --- | --- | --- | --- |
| IL-1β | -.099 | -.310 | -.244 | .003 | .081 | .126 | .020 |
| IL-4 | -.042 | -.194 | .276 | -.376* | -.131 | -.457* | -.445* |
| IL-6 | -.068 | .063 | -.143 | .075 | .010 | -.077 | -.042 |
| IL-8 | .054 | .092 | -.128 | .157 | .005 | -.051 | -.076 |
| IL-10 | .054 | .360 | -.062 | .039 | -.013 | .023 | .031 |
| IL-12 | .035 | .337 | -.092 | -.016 | -.038 | .027 | .065 |
| IL-17 | .144 | .111 | .037 | .001 | -.393* | .233 | .201 |
| IFN-γ | .168 | .329 | .180 | -.144 | -.138 | -.089 | -.061 |
| MCP-1 | .042 | .125 | -.129 | .116 | .033 | -.058 | -.067 |
| MIP-1β | .060 | .351 | -.064 | .016 | -.021 | .012 | .027 |
| TNF-α | -.069 | -.038 | .182 | -.230 | -.255 | -.228 | -.081 |

A positive value indicates a positive correlation, whereas a negative value indicates a negative correlation, * < 0.05. Abbreviations: IL interleukin, G-CSF granulocyte colony-stimulating factor, IFN interferon, MCP monocyte chemoattractant protein, MIP macrophage inflammatory protein, TNF tumour necrosis factor, CRP C reactive protein.

**Table 3** Correlation coefficients between parameters of circulation and concentrations of cytokines and chemokines in SEBC

| Cytokine/chemokine | MAP | UO | Serum lactate | Noradrenaline dose | pH |
| --- | --- | --- | --- | --- | --- |
| IL-1β | -.366 | -.070 | .889** | .316 | -.313 |
| IL-4 | .293 | -.079 | .288 | .100 | .278 |
| IL-6 | -.339 | .034 | .817** | .380 | -.079 |
| IL-8 | -.305 | .113 | .742* | -.093 | .076 |
| IL-10 | -.255 | -.112 | .321 | .521 | .022 |
| IL-12 | -.259 | -.067 | .076 | .514 | -.001 |
| IL-17 | .048 | .261 | .110 | -.184 | .170 |
| IFN-γ | -.268 | -.097 | .096 | .559* | .091 |
| MCP-1 | -.300 | .112 | .233 | .626* | .068 |
| MIP-1β | -.263 | -.102 | .797** | .518 | .029 |
| TNF-α | -.427* | -.272 | .790** | .389 | -.195 |

A positive value indicates a positive correlation, whereas a negative value indicates a negative correlation, * < 0.05, **p<0.01. Abbreviations: IL interleukin, G-CSF granulocyte colony-stimulating factor, IFN interferon, MCP monocyte chemoattractant protein, MIP macrophage inflammatory protein, TNF tumour necrosis factor, MAP mean arterial pressure, UO urinary output.
